# Reward Prediction Errors Drive Reinforcement Learning and Recognition Memory: Gender Differences in Methamphetamine Abusers

**DOI:** 10.1002/brb3.71630

**Published:** 2026-09-14

**Authors:** YuQing Liu, Die Wang, LePing Wang, SongSong Wang, ZhiYing Chen, Tiancheng Wang, Shengyi Jin, Qiang Zhou, Liping Yang

**Affiliations:** ^1^ Department of Psychology Wenzhou Medical University Wenzhou China; ^2^ Psychological Assessment Unit, Department of Psychiatry The Second Affiliated Hospital & Yuying Children's Hospital of Wenzhou Medical University Wenzhou China; ^3^ School of Psychology Shandong Normal University Jinan China; ^4^ The Center for Geriatric Medicine The First Affiliated Hospital of Wenzhou Medical University Wenzhou China; ^5^ Zhejiang Moganshan Female Compulsory Isolation Drug Rehabilitation Center Huzhou China; ^6^ Wenzhou Huanglong Compulsory Isolation Drug Rehabilitation Center Wenzhou China; ^7^ Wenzhou Ouhai District Anti‐Drug Committee Office Wenzhou China; ^8^ Lishui Second People's Hospital Affiliated to Wenzhou Medical University Lishui China

**Keywords:** executive functioning, methamphetamine, recognition memory, reinforcement learning, reward prediction errors

## Abstract

**Objectives:**

Methamphetamine (MA) abusers have impaired executive functioning with gender differences, which may be related to the reward prediction errors (RPEs) that drive reinforcement learning and recognition memory, as RPE signaling is impaired in MA abusers. We examined whether the driving effects of RPE on reinforcement learning and recognition memory are impaired in MA abusers and how they differ between women and men.

**Methods:**

Eighty‐two MA abusers (43 men, 39 women) from Zhejiang Compulsory Isolation Drug Rehabilitation Center and 86 non‐users (45 men, 41 women) from Wenzhou medical university were recruited in this experimental study. We employed a behavioral paradigm adapted from Rouhani et al., which included a learning task and a recognition test. In the learning task, participants underwent a cycle of prediction and feedback, using the outcomes to iteratively adjust their subsequent choices. Reward prediction errors were manipulated by giving different monetary reward feedback after each reward prediction.

**Results:**

MA abusers showed significant impairments in learning rates and recognition accuracy compared with non‐users. GLMM analysis revealed gender‐specific patterns: the moderating effect of group on the RPE‐learning relationship was significant in females but not in males, indicating impaired RPE utilization in female abusers.

**Conclusion:**

These findings suggest that methamphetamine abuse impairs RPE‐dependent learning and memory, with reduced RPE utilization efficiency specifically observed in female abusers—manifested as larger prediction errors but lower learning rates.

## Graphical abstract

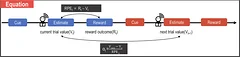

## 1 Introduction

Methamphetamine (MA) abusers generally exhibit impaired executive functions, particularly in domains of complex decision‐making and cognitive flexibility (Park et al. 2010; Mizoguchi and Yamada 2019). This cognitive deficit contributes to their difficulty in quitting drug use (Cox et al. 2016). Specifically, even when drug use yields no positive or even negative outcomes, MA abusers struggle to integrate past experiences flexibly and make adaptive decisions to abstain (Salo et al. 2013). Instead, they tend to prioritize short‐term benefits, seeking immediate gratification through continued drug use (Kalivas and Volkow 2005). Given that addiction arises from interactions among neural circuits governing executive control, reward processing, and learning (Volkow and Fowler 2000), a key question emerges: is this impairment in executive functioning linked to aberrant reward learning mechanisms—specifically, those based on reward prediction errors—in MA abusers (Hauser et al. 2015)?

Reward prediction errors (RPEs) refer to the differences between predicted and received rewards (Schultz et al. 1997). RPEs are a crucial factor in reinforcement learning and memory, and they play a vital role in everyday learning and flexible decision‐making (Hauser et al. 2015; Collins et al. 2017). The Rescorla–Wagner model (Rescorla and Wagner 1972) argued that individuals learn the value of events through dynamically changing RPEs rather than simply repeating rewards. The subsequent temporal difference reinforcement learning model (Daw and Touretzky 2003; Sutton and Barto 2018) emphasized that RPE‐driven reinforcement learning is a dynamic process: individuals make repeated attempts and mistakes under multiple stimulus‐reward scenarios, updating expectations and then engaging in behavioral changes (O'Doherty 2012; Wang et al. 2018; Schultz 2016).

The role of RPEs in memory has recently begun receiving attention. Also, there are multiple ways in which RPEs influence memory (Jang et al. 2019; Sinclair et al. 2021; Gan et al. 2025). Furthermore, feedback that is different from expectation can enhance recognition memory, which can then provide individuals with previous information in new scenarios, guiding their future choices (Rouhani and Niv 2021). That is, the reward expectation obtained from the reward learning process and memories of the previously experienced results jointly influence individuals’ decision‐making (Yazin et al. 2021). However, research on how reward prediction errors (RPEs) coordinate the interaction between reward learning and recognition memory remains limited in clinical populations such as methamphetamine abusers. To address this gap, the present study employed a behavioral paradigm capable of simultaneously assessing reinforcement learning and recognition memory (Rouhani et al. 2018), aiming to test the core hypothesis that methamphetamine abuse disrupts the adaptive guidance of behavior by RPE‐enhanced recognition memory. Specifically, we propose that if this mechanism is impaired, individuals would exhibit diminished capacity to utilize past feedback to guide subsequent decisions, leading to persistent maladaptive behavioral patterns despite negative outcomes.

RPE signaling is encoded by dopamine neurons. During the learning of natural rewards, dopamine neurons in the brain are activated when the outcome does not match expectations (Schultz 2013; Starkweather and Uchida 2021; Montague et al. 2004). Afterward, the dopamine‐encoded RPEs (DA‐RPEs) signals are transmitted to the downstream regions of the brain and modulate plasticity in the striatum and hippocampus, which drive reinforcement learning and memory of current events, respectively (Lisman et al. 2011). However, methamphetamine (MA) is a stimulant that readily penetrates the central nervous system and acts like natural rewards by activating the dopamine system, thereby generating excessive false DA‐RPE signals. Repeated exposure to this dopamine surge leads to diminished neurological sensitivity to natural rewards (Nestler 2004; Torregrossa et al. 2011), consequently impairing the processing of natural reward‐based prediction errors in MA abusers (Bernacer et al. 2013).

In addition, some researchers suggest that methamphetamine has a stronger rewarding and motivating effect on females which leads them to show stronger dopamine neuron activation than males and that abnormal RPE signal reinforces habitual drug use, resulting in more cognitive and social function impairment in females (Anker and Carroll 2011; Ballard et al. 2015; Towers et al. 2021). In addition, studies on gender differences in executive functioning have found that both decision‐making and cognitive flexibility may be more impaired in female MA abusers than in males (van der Plas et al. 2009). Based on these, there may be sex differences in the driving effects on reinforcement learning and recognition memory in MA abusers.

This study aims to investigate whether the driving effects of RPEs on reinforcement learning and recognition memory were impaired in MA abusers and how differ across genders. Reinforcement learning and recognition memory were investigated concurrently (Rouhani et al. 2018). Following the reward prediction paradigm, the participants’ predicted and actual reward feedback values were accompanied by the presentation of a novel pictorial stimulus each time. RPEs were manipulated by giving the participants different monetary reward feedback after each reward prediction. The purpose was to examine the trends in reinforcement learning and recognition memory generated by RPE value fluctuations.

## 2 Methods

### 2.1 Participants

The G*Power software (version 3.1.9.7) was used, with effect size and significance level set to 0.15 and 0.05, respectively. A total of 180 participants were recruited in this experiment. Ninety participants with MA use disorder (MA abusers) were recruited from Zhejiang Compulsory Isolation Drug Rehabilitation Center, of which 45 females came from Moganshan Women's Drug Rehabilitation Center and 45 males came from Huanglong Drug Rehabilitation Center. The following were the criteria for inclusion: (1) meeting diagnostic criteria for MA use disorder and addiction in DSM‐IV; (2) had undergone rehabilitation for more than one month; (3) had educational levels of junior high school and above; (4) no co‐morbid mental retardation; (5) no addiction to substances other than MA; (6) no history of depression or other mental illness; and (7) no serious chronic diseases, such as brain injury, cardiovascular disease, chronic diseases, etc. The 90 non‐users (45 men and 45 women) were recruited from the community.

After the preliminary check, the following data were excluded: (1) incomplete data collection and (2) accuracy rate below 50%. Eighty‐two MA abusers (*M* = 32.37 years, *SD* = 7.39; 43 men, 39 women) and 86 non‐users (*M* = 19.86 years, *SD* = 1.99; 45 men, 41 women) were included.

This study was conducted by the Declaration of Helsinki and was approved by the Ethics Committee of Wenzhou Medical University. All participants volunteered to participate in the study and signed informed consent forms.

### 2.2 Materials

The experiment involved 80 geometric images, and all the images were standardized using Photoshop. Sixty‐four images were used for the feedback phase, and each of them was randomly assigned a reward value (30, 35, 40, 45, 55, 60, 65, or 70 points). The images for memory testing consisted of 32 randomly selected images, of which 16 were old (appeared during the learning phase) and 16 were new. The stimuli were presented using Psychopy 3.0 on a 17‐inch screen with a resolution of 1024 × 768 and a refresh rate of 60 Hz.

### 2.3 Procedure

**FIGURE 1:**
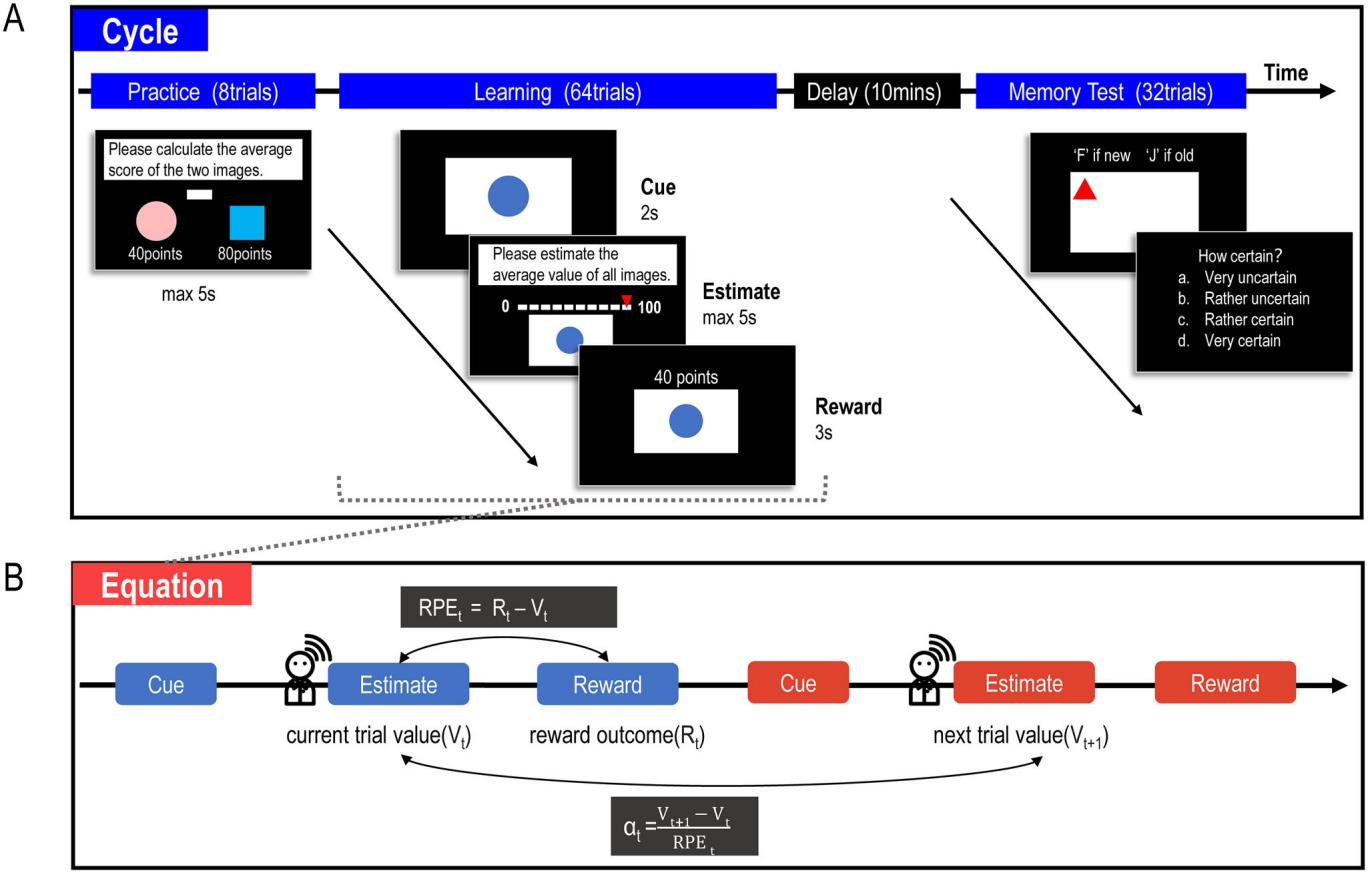
Experimental design. (A) Practice task. Participants were asked to calculate the average value of the two images presented and enter the average value into the answer box. Learning task. Participants were shown an image (cue), then were asked to estimate the average reward value for all images (estimate). Finally, the reward value of the image is presented to the participants (reward). Memory test, participants were asked to judge whether the picture was new or old and then choose the certainty of the answer. (B) In the two consecutive trials of the learning phase, we calculated the RPE and learning rate by formula. RPE*_t_* = *R_t_* – *V_t_*, the reward prediction error is the difference between the estimated reward and the reward feedback value in the same trial. *α_t_* = (*V_t_*_+1_ – *V_t_*)/RPE *_t_*, the learning rate is the ratio of the difference between the estimated reward in consecutive trials and the reward prediction error of the previous trial.

A two‐factor mixed experimental design was adopted after referring to the reward prediction paradigm of Rouhani et al. (2018), including three phases: Practice, Learning, and Memory test (Figure 1). The between‐subject variable was the group (MA abusers/non‐users) and the within‐subject variable was the RPEs (ranging from –70 to 70). The dependent variables were the learning rate of the geometric images (0–1) and the rate of accuracy at recognizing the geometric images (correct/wrong).

#### 2.3.1 Practice

Participants were asked to complete eight practice trials and calculate the average reward value of the images to ensure that they had sufficient cognitive level to participate in the experiment.

#### 2.3.2 Learning

During each of the 64 trials, a geometric image was present for 2 s, after which participants had 5 s to estimate the average value of the image (1–100). Next, that image and its actual reward value were shown on the screen, achieving the reward feedback effect. Images were randomly assigned actual reward values and presented once. Before the experiment, participants were informed that each estimate should reflect the average value of all images and that the actual reward feedback presented in each trial only represented the value assigned to the specific image.

The difference between the predicted and actual average reward values was defined as a single RPE. After experiencing the difference between the predicted and actual average reward values for each image, participants used previous errors to continuously adjust their estimates. This was the same process as reinforcement learning.

#### 2.3.3 Memory

After a 10‐min break, recognition memory was tested in 32 trials. On each trial, participants were asked to indicate whether a geometric image had appeared previously (yes/no) and to rate their confidence in this judgment on a 4‐point scale (1 = complete certainty, 4 = complete guesswork).

Recognition accuracy was defined as the hit rate for old images—the number of old images correctly identified as “old” divided by the total number of old images (16). Responses to new images were not included in the main analysis; they were used solely to calculate false alarm rates. For the confidence scale, a correct response was defined as one in which the participant correctly identified the old image and did not select “complete guessing” (4). If a participant responded correctly but gave a confidence rating of 4, the trial was excluded from the accuracy calculation. This criterion was applied to ensure that recognition accuracy reflected genuine memory retrieval rather than random guessing. This stage was designed to verify participants’ recognition accuracy and to examine whether RPE values associated with the images influenced accuracy rates.

### 2.4 Statistical Analysis

SPSS 24.0 was used for data analysis. As an indicator for reward prediction errors, absolute prediction error is the unsigned prediction error. |PE*_t_* | = |*R_t_* – *V_t_* |, where *R_t_* and *V_t_* refer to the feedback and predicted values of a single reward respectively. The absolute value of the reward feedback value (*R_t_*) minus the reward prediction value (*V_t_*) of each trial is obtained as the absolute prediction error of a single participant (Rouhani and Niv 2021). An ANCOVA was used to compare the reward prediction errors (|RPEs|) in different groups, with Group and Gender as independent variables, age as covariate.

Then, ANOVAs and GLMMs were used to analyze how Gender and Group effected the effect of RPE on reinforcement learning and recognition memory. In reinforcement learning, the current reward feedback value (*R_t_*) was used to update the current reward prediction (*V_t_*) and obtain the next reward prediction (*V_t_*_+ 1_), so the learning rate *α_t_* = (*V_t_*_+ 1_ – V_t_)/(*R_t_* – *V_t_*) (Rouhani and Niv 2021). The recognition accuracy rate was used to measure recognition memory. For those, we first conducted an analysis of covariance (ANCOVA) to examine group differences in reinforcement learning and recognition memory, with Gender and Group as independent variables and age as a covariate. Subsequently, we employed two generalized linear mixed models (GLMMs) for further analysis. These models used learning rates and recognition accuracy as dependent variables, respectively, with |RPEs| and Group as fixed factors. This approach was designed to test whether the effects of these factors varied significantly by gender.

## 3 Results

### 3.1 RPE Analysis

No significant main effect of Group was found for |PEt| (*F* = 0.939, *p* = 0.334, *ƞ* ^2^ = 0.006), nor for Gender (*F* = 1.087, *p* = 0.299, *ƞ* ^2^ = 0.007). Critically, the Group × Gender interaction was significant (*F =* 13.126*, p* < 0.001*, ƞ* ^2^ *=* 0.077). Simple effects analysis revealed that female MA abusers exhibited significantly larger absolute prediction errors than female non‐users (*F =* 11.053*, p* = 0.001*, ƞ* ^2^ *=* 0.066), whereas no significant difference was found between the male groups (*F =* 0.882*, p* = 0.349*, ƞ* ^2^ *=* 0.006). These results indicate that the overall group difference was driven primarily by the female subgroup, with female MA abusers showing significantly less accurate reward estimation than female non‐users, while no such effect was observed in males (Table 1).

**TABLE 1:** Descriptive statistics.

	Group Male MA group (*n* = 43)	Male control group (*n* = 45)	Female MA group (*n* = 39)	Female control group (*n* = 41)
Age (years)	37.05 ± 6.02	20.63 ± 2.16	27.08 ± 3.06	19.03 ± 0.88
Years of drug use	8.20 ± 4.96		4.41 ± 2.53	
|RPE|	18.03 ± 13.68	19.87± 15.28	21.76 ± 15.21	16.60± 10.60
Learning rate	0.153 ± 0.29	0.18 ± 0.31	0.17 ± 0.31	0.27 ± 0.36
Recognition memory	0.80 ± 0.39	0.80 ± 0.40	0.64 ± 0.48	0.81 ± 0.39

*Note*: This table presents the learning rates and recognition memory of the MA group and non‐users’ group.

### 3.2 Reinforcement Learning

Analysis of learning rates revealed significant main effects of both Group (*F* = 8.368, *p* = 0.004, *ƞ* ^2^ = 0.051) and Gender (*F* = 7.739, *p* = 0.006, *ƞ* ^2^ = 0.047). MA abusers demonstrated significantly lower learning rates than non‐users, and males showed lower learning rates than females across groups (Table 1). A significant Group × Gender interaction was observed (*F* = 12.485, *p* < 0.001, *ƞ^2^* = 0.074).

**FIGURE 2:**
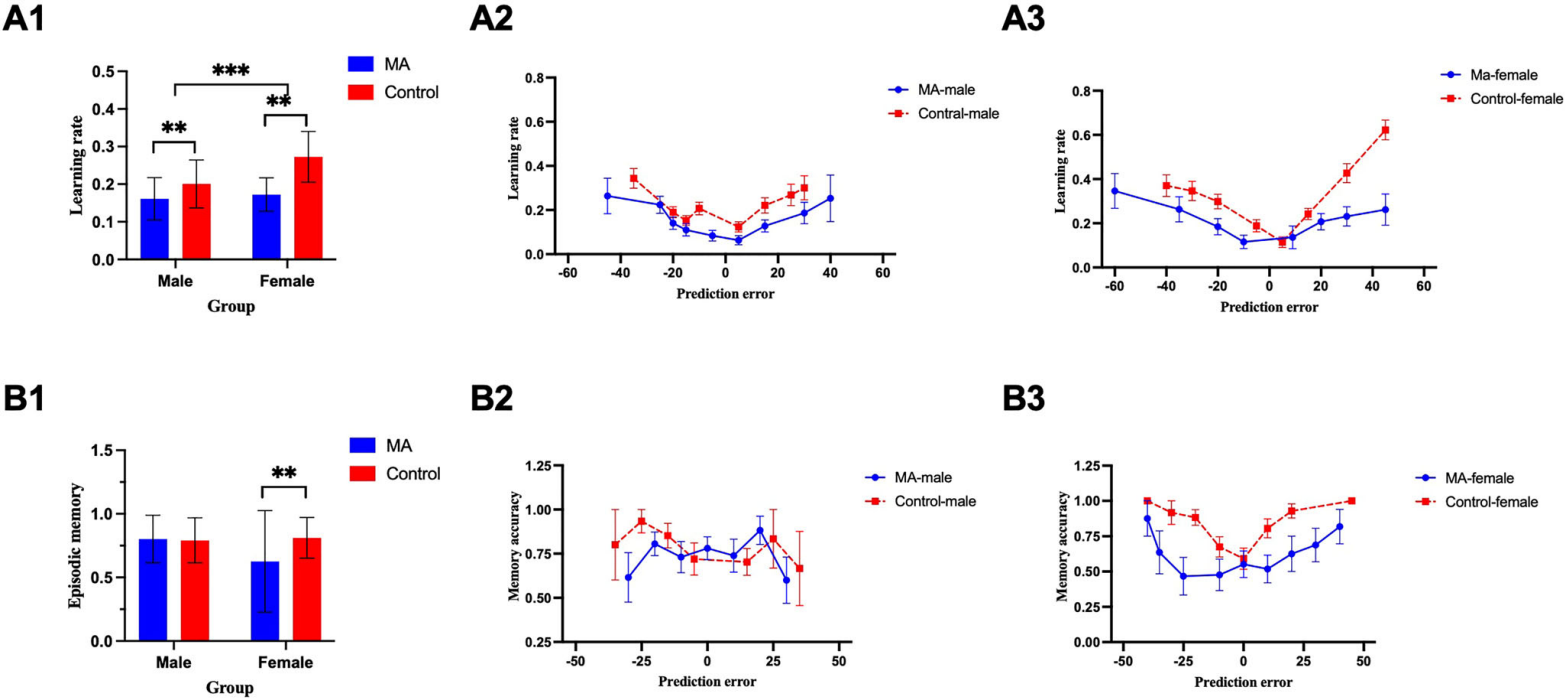
Experimental results. (A) Results of test of learning rates: (A1) comparison of learning rates of four subject types as driven by RPE; (A2) average learning rates corresponding to prediction errors by two male subject types; and (A3) average learning rates corresponding to prediction errors by two female subject types. (B) Results of test of learning rates: (B1) comparison of memory accuracy of four subject types as driven by RPE; (B2) average memory accuracy corresponding to prediction errors by two male subject types; and (B3) average memory accuracy corresponding to prediction errors by two female subject types. ^*^ *p* < 0.05, ^**^ *p* < 0.01, ^***^ *p* < 0.001.

Simple effects analysis demonstrated that MA abusers had significantly lower learning rates than non‐users in females (*F* = 27.666, *p* < 0.001, *ƞ* ^2^ = 0.150), but not in males (*F* = 0.273, *p* = 0.602, *ƞ* ^2^ = 0.002) (see Figure 2A, 1). This pattern—a large and significant effect in females versus a negligible and non‐significant effect in males—indicates that the significant Group × Gender interaction (*F* = 12.485, *p* < 0.001, *η^2^* = 0.074) reflects a substantially larger MA‐induced learning rate deficit in females than in males. Further analysis revealed no significant gender difference in learning rates among MA abusers (*F* = 0.085, *p* = 0.771, *ƞ* ^2^ = 0.001). In contrast, among non‐users, males exhibited significantly lower learning rates than females (*F* = 27.732, *p* < 0.001, *ƞ* ^2^ = 0.150).

To further investigate these effects, we conducted separate GLMMs for male and female participants with Group and absolute RPE as fixed effects. For female participants, the analysis revealed significant main effects of Group (*β* = –0.382, *t* = –3.837, *p* < 0.001) and absolute RPE (*β* = –0.026, *t* = –4.253, *p* < 0.001). Critically, the Group × absolute RPE interaction was also significant (*β* = 0.020, *t* = 3.232, *p* = 0.002), indicating that the relationship between absolute prediction errors and learning rates was modulated by group membership. Specifically, the negative association between |RPE| and learning rate was weaker in the MA group (*β* = –0.006) than in the control group (*β* = –0.026), suggesting a potentially impaired ability to utilize RPE signals for learning in female MA abusers (Figure 2A3).

For male participants, the GLMM revealed a significant main effect of absolute RPE (*β* = –0.004, *t* = –2.009, *p* = 0.048), but no significant main effect of Group (*β* = –0.093, *t* = –1.744, *p* = 0.085) or Group × absolute RPE interaction (*β* = 0.003, *t* = 1.040, *p* = 0.302; Figure 2A2).

These results demonstrate that the relationship between absolute prediction error and reinforcement learning was moderated by Group in female but not male MA abusers.

### 3.3 Recognition Memory

Analysis of recognition accuracy revealed a significant main effect of Group (*F* = 5.993, *p* = 0.015, *ƞ* ^2^ = 0.037), with MA abusers demonstrating lower accuracy than controls. The main effect of Gender was not significant (*F* = 0.747, *p* = 0.389, *ƞ* ^2^ = 0.005). No significant Group × Gender interaction was observed (*F* = 2.324, *p* = 0.129, *ƞ* ^2^ = 0.015).

Simple effects analysis revealed no significant gender difference in recognition accuracy within either the MA group (*F* = 2.153, *p* = 0.144, *η^2^* = 0.014) or the control group (*F* = 0.215, *p* = 0.643, *η^2^* = 0.001). When examining gender‐specific group differences, recognition accuracy was significantly lower in female MA abusers compared to female controls (*F* = 12.338, *p* < 0.001, *ƞ* ^2^ = 0.073), while no significant difference was observed between male MA abusers and male controls (*F* = 1.236, *p* = 0.268, *ƞ* ^2^ = 0.008; Figure 2B, 1).

To further examine whether the observed group differences in recognition accuracy were driven by RPE signals, separate GLMMs were conducted. For females, no significant main effect of absolute RPE was observed (*β* = 0.022, *t* = 1.388, *p* = 0.169). The main effect of Group was also not significant (*β* = –0.188, *t* = –0.600, *p* = 0.550), nor was the absolute RPE × Group interaction (*β* = –0.006, *t* = –0.322, *p* = 0.748; Figure 2B3). For males, the GLMM showed no significant main effect of absolute RPE (*β* = 0.004, *t* = 0.572, *p* = 0.569), no significant main effect of Group (*β* = –0.017, *t* = –0.100, *p* = 0.920), and no significant Group × absolute RPE interaction (*β* = –0.001, *t* = –0.113, *p* = 0.911; Figure 2B2).

These results indicate that absolute prediction errors did not significantly enhance recognition memory in either males or females, nor did they modulate the observed group differences in recognition accuracy.

## 4 Discussion

The present study investigated the effects of methamphetamine (MA) addiction on reward prediction error (RPE)‐based learning and memory functions through behavioral approaches, while also revealing gender differences in their underlying mechanisms. The main findings can be summarized as follows: At the behavioral level, gender‐specific patterns of cognitive impairment emerged. Female MA abusers exhibited significantly larger absolute prediction errors (|RPE|) and significantly lower learning rates and recognition memory accuracy than female controls, whereas male MA abusers showed significantly lower learning rates but comparable |RPE| and recognition memory accuracy relative to male controls. Collectively, these results indicate that methamphetamine abuse indeed impairs core cognitive processes dependent on RPE signaling (Salo et al. 2009; Groman et al. 2022; Salas‐González et al. 2024).

Notably, this study further elucidated gender‐specific patterns of cognitive impairment through generalized linear mixed models (GLMM). We found that although larger absolute prediction errors (|RPE|) significantly drove reinforcement learning in both control and MA abusers’ groups (including both genders)—consistent with the theory that “unexpected reward feedback promotes learning” (Pine et al. 2018)—there were significant gender differences in its driving efficiency. This paradoxical phenomenon constitutes a key finding of our study: although female MA abusers exhibited larger absolute prediction errors, their learning rates were significantly lower than those of female non‐users, suggesting a specific impairment in this population's ability to translate unexpected reward feedback into behavioral adjustments.

From a neurobiological perspective, we speculatively suggest that this gender‐specific impairment may be associated with differential adaptations in the dopamine system. Repeated MA exposure induces excessive dopamine release (Torregrossa et al. 2011; Nestler 2004), which may lead to reduced neural sensitivity to natural rewards in female abusers, potentially impairing their natural reward‐based RPE signal processing (Bernacer et al. 2013). Consequently, although the “signal” (|RPE|) is amplified, the brain's capacity to utilize this signal to guide learning is compromised, ultimately manifesting as decreased learning rates. This phenomenon of “strong signal but low efficiency” may reflect the essential characteristic of impaired learning mechanisms in female MA abusers. However, given the purely behavioral nature of the present study, this interpretation remains speculative and lacks direct neural evidence. Future research employing neuroimaging techniques (e.g., fMRI) is necessary to directly validate the hypothesized dopaminergic mechanisms.

In contrast, male MA abusers exhibited a distinct pattern: their RPE signal strength showed no significant difference from the control group, and the driving effect of this signal on reinforcement learning remained intact, indicating relatively preserved RPE‐based learning mechanisms. This gender difference highlights the heterogeneity in cognitive impairment mechanisms associated with MA abuse, providing new perspectives for understanding gender disparities in addictive behaviors (Mayo et al. 2019; Armenta‐Resendiz et al. 2024).

The present study, through behavioral experiments and methodological approaches, provides evidence for extensive impairment of RPE‐mediated learning and memory in MA abusers but, more importantly, suggests that these impairments are modulated by gender. For female MA abusers, the core issue lies in the impaired utilization efficiency of RPE signals, a finding with significant clinical implications. It suggests that future cognitive‐behavioral interventions should consider gender factors, potentially requiring particular emphasis on rebuilding the ability to translate unexpected feedback into behavioral adjustments for female abusers.

It is worth noting that the task in this study required participants to estimate the mean reward value of all images on each trial, a process that likely engages working memory (WM) resources. Given that WM deficits in methamphetamine (MA) abusers have been widely reported (e.g., Potvin et al. 2018; Yao et al. 2024), the observed reduction in learning rates may be partially attributable to increased WM load rather than purely reflecting impaired RPE signal utilization efficiency. Future studies should therefore consider minimizing WM load in experimental designs to more cleanly isolate RPE‐driven learning mechanisms, or include direct measures of WM capacity (e.g., digit span or N‐back tasks) to statistically control for individual differences in WM, thereby helping to disentangle the contributions of WM from those of RPE processing.

In addition, the recognition memory test included only 16 trials. Given the approximately normal distribution of learning‐phase |RPE|, with few high‐RPE events, this limited set may not have adequately sampled such events, potentially constraining sensitivity to detect RPE‐driven memory effects. Future studies should increase test trials to more robustly examine the RPE‐memory relationship.

This study has several limitations. A notable limitation of this study is the significant age difference between groups (MA group: 32.4 years; control group: 19.9 years). Age is an important correlate of cognitive function, and although we statistically controlled for it, this bias cannot be fully eliminated. Nevertheless, our core conclusions appear to remain robust. If age were the main cause of the observed cognitive differences, older individuals would be expected to perform worse on cognitive measures. However, the opposite pattern emerged: male MA abusers, despite being older, showed significantly better recognition memory than female MA abusers and performed comparably to male controls. This indicates that age alone cannot explain the specific impairments observed in female MA abusers. The fact that younger female abusers exhibited the most severe deficits further suggests that MA abuse has a genuine and gender‐specific impact on RPE processing. Thus, while age mismatch is a limitation, it does not undermine the main conclusions of this study. Future studies should employ age‐matched control groups.

Additionally, all methamphetamine abusers in this study were in a compulsory abstinence period, and only years of drug use were reported, without information on abstinence duration, frequency, or route of administration. These missing variables may affect cognitive performance and limit the generalizability of our findings. Future studies should include or at least describe these variables in detail and consider individuals at different stages of abstinence or those still actively using, to more comprehensively assess the effects of methamphetamine abuse on cognitive function.

## Author Contributions

**Die Wang**: writing – original draft, conceptualization, methodology, writing – review and editing, data curation. **SongSong Wang**: writing – review and editing. **YuQing Liu**: conceptualization, investigation, writing – original draft, methodology, writing – review and editing, data curation, supervision. **Shengyi Jin**: resources. **ZhiYing Chen**: resources. **Tiancheng Wang**: resources. **LePing Wang**: software, data curation, formal analysis, writing – review and editing. **Qiang Zhou**: supervision, conceptualization, methodology, writing – original draft, writing – review and editing, funding acquisition, resources. **Liping Yang**: writing – review and editing, supervision.

## Funding

This work was supported by grants from the National Social Science Fund of China (Code: 20BSH047).

## Ethics Statement

This study was approved by the Ethics Committee of Wenzhou Medical University (2022‐016) and conducted in accordance with the Declaration of Helsinki. All participants wrote informed consent prior to completion of the research and their independent will was emphasized.

## Conflicts of Interest

We declare that we have no financial and personal relationships with other people or organizations that can inappropriately influence our work; there is no professional or other personal interest of any nature or kind in any product, service, and/or company that could be construed as influencing the position presented in, or the review of, the manuscript entitled.

## Data Availability

Data supporting the findings of this study are available from the authors at https://github.com/YuqingLiu666/Supplementary‐Materials
